# Indirect costs related to caregivers’ absence from work after paediatric tonsil surgery

**DOI:** 10.1007/s00405-017-4526-7

**Published:** 2017-03-14

**Authors:** Gunnhildur Gudnadottir, G. Ragnarson Tennvall, J. Stalfors, J. Hellgren

**Affiliations:** 1 Department of Otorhinolaryngology, Sahlgrenska University Hospital, Sahlgrenska Academy, Gothenburg University, Gröna stråket 9, 413 46 Gothenburg, Sweden; 2 The Swedish Institute for Health Economics (IHE), Lund, Sweden

**Keywords:** Tonsillectomy, Tonsillotomy, Absenteeism, Indirect cost, Health economy

## Abstract

Tonsillotomy has gradually replaced tonsillectomy as the surgical method of choice in children with upper airway obstruction during sleep, because of less postoperative pain and a shorter recovery time. The aim of this study was to examine the costs related to caregivers’ absenteeism from work after tonsillectomy (TE) and tonsillotomy (TT). All tonsillectomies and tonsillotomies in Sweden due to upper airway obstruction during 1 year, reported to the National Tonsil Surgery Register in children aged 1–11 were included, *n* = 4534. The number of days the child needed analgesics after surgery was used as a proxy to estimate the number of work days lost for the caregiver. Data from the Social Insurance Agency (Försäkringskassan) regarding the days the parents received temporary parental benefits in the month following surgery were also analysed. The indirect costs due to the caregivers’ absenteeism after tonsillectomy vs tonsillotomy were calculated, using the human capital method. The patient-reported use of postoperative analgesic use was 77% (*n* = 3510). Data from the Social Insurance Agency were gathered for all 4534 children. The mean duration of analgesic treatment was 4.6 days (indirect cost of EUR 747). The mean number of days with parental benefits was 2.9 (EUR 667). The indirect cost of tonsillectomy was 61% higher than that of tonsillotomy (EUR 1010 vs EUR 629). The results show that the choice of surgical method affects the indirect costs, favouring the use of tonsillotomy over tonsillectomy for the treatment of children with SDB, due to less postoperative pain.

## Introduction

Sleep-disordered breathing (SDB) is a common disorder in children, affecting 4–11% with symptoms of snoring, apneas and choking sounds during sleep [1]. Tonsillectomy with or without adenoidectomy (TE ± A) has been the treatment of choice for children with sleep-disordered breathing due to adenotonsillar hypertrophy. In recent years, in Sweden, tonsillotomy (TT) has replaced tonsillectomy as the recommended treatment for children with SDB due to tonsillar hypertrophy [2]. Studies have shown a lower risk of postoperative bleeding and less pain after tonsillotomy compared with tonsillectomy [3, 4]. Tonsillectomy is still, however, the preferred method of surgery for chronic or recurrent tonsillitis. Tonsil surgery is one of the most common surgical procedures performed in children under general anaesthesia, with more than 500,000 procedures performed annually in the USA [5].

The benefits of treating children with SDB surgically have been shown in a number of studies of general well-being, quality of sleep and cognitive function [6, 7]. However, less has been published about the health-economic impact of surgery for SDB in children.

Children with SDB have been shown to have considerably higher health-care use compared with other children [8] and Tarasiuk et al. reported that the high costs due to health-care use in children with SDB dropped by 30%, 1 year after adenotonsillectomy, mainly due to fewer respiratory infections [9]. Chang et al. obtained similar results when comparing Medicaid costs 2 years prior to and 2 years after a tonsillectomy in children with adenotonsillar hypertrophy, although the decrease in costs over the 2 years did not compensate for the cost of surgery in that study [10]. To our knowledge, there are no previous studies of the health-economic costs of caregivers’ absence from work, also known as absenteeism, while caring for their children after surgery for SDB. Caregiver absenteeism generates costs that are not primarily borne by the health-care system but by society as a whole in the form of lost production. While the costs of surgery, hospital visits and medication are usually described in terms of direct costs, caregivers’ absenteeism from work is referred to as indirect costs.

To estimate the total costs associated with SDB in children, it is important to consider both the direct and indirect costs. The direct costs of tonsil surgery are likely to vary considerably between health-care systems and countries and will be influenced by market competition and the ability for correct pricing in complex hospital economies [11–13]. Factors affecting indirect costs, such as absence from work, are, however, specifically related to the disease, such as duration of pain after surgery or inability to feed after surgery, and are, therefore, likely to be comparable between countries and health-care systems.

The aim of this study was to assess the indirect costs related to caregivers’ absenteeism due to tonsil surgery in children with SDB and whether these indirect costs differ between tonsillectomy and tonsillotomy. For this purpose, data from the National Tonsil Surgery Register in Sweden (NTSRS) regarding caregiver-reported use of analgesics after surgery were collected for all paediatric tonsil surgeries during 1 year. This information was used as a proxy for the days the children needed to be cared for at home. Data were also collected from the Swedish Social Insurance Agency (Försäkringskassan) regarding days with temporary parental benefits for all caregivers in the month following surgery.

## Materials and methods

The indirect costs of caregivers’ absenteeism in relation to their children’s tonsil surgery due to SDB were calculated using data from the National Tonsil Surgery Register Sweden (NTSRS) and the Swedish Social Insurance Agency. In 2011, the NTSRS included 77.5% of all tonsil surgeries performed in Sweden in 2011 and all registered children aged 1–11 with the main indication upper airway obstruction were included in the study. The indirect costs of the four surgical procedures applied in SDB, tonsillectomy (TE), adenotonsillectomy (TE + A), tonsillotomy (TT), and tonsillotomy with adenoidectomy (TT + A), were compared. The indication for tonsillotomy according to the national Swedish guidelines is children with sleep disordered breathing and enlarged tonsils [14]. There is no specific age limit for choosing tonsillectomy over tonsillotomy or vice versa. If the main indication is recurrent infections, tonsillectomy is preferred, however, there is no absolute contraindication against doing tonsillotomy in these children. In the NTSRS the physician registers the main indication for surgery, and the cohort selected for this study were patients operated with upper airway obstruction. Accordingly, the study population can include children with both obstruction and recurrent infections though the vast majority would have suffered from obstruction only.

### NTSRS data

The NTSRS is administered by the Swedish Association for Otorhinolaryngology, Head & Neck Surgery (http://www.orlforum.se) and funded by the Swedish Association of Local Authorities and Regions, SKL (http://www.skl.se). The register was founded in 1997 and the purpose of the register is to monitor and reinforce the quality of Swedish health care. In the present study, we have used data on tonsil surgery due to SDB in all children aged 1–11 years (up to their 12th birthday), reported to the register in 2011. The surgeries included are TE, TE + A, TT and TT + A. The NTSRS collects data from both professionals and patients through questionnaires. The NTSRS collects data on age, gender, type of care, indication for surgery, technique for dissection, technique for haemostasis and postoperative haemorrhage, as well as patient-related outcome measurements of postoperative pain, postoperative infections, symptom relief and how the information provided regarding surgery was collected [3]. In the present study, the following data from the NTSRS were used: type of surgery (TE, TE + A, TT, TT + A) reported by the professionals and patient-/caregiver-reported number of days using analgesics from the questionnaire sent to patients 30 days after surgery. The reported number of days with analgesics was used to estimate a minimum number of days a caregiver would have to stay at home after surgery. The validity of this approach is based on the fact that, in Sweden, more than 85% of children aged 1–5 are enrolled at day-care centres until pre-school at 6 years of age. Medication, including analgesics, is not given to children by the staff at day-care centres or schools in Sweden. When in need of regular medication after surgery, the children would, therefore, have to stay at home. Sweden has a high employment rate for both genders, as around 80% of the population aged 20–64 are employed. Normally, both parents work or study and the level of employment among Swedish women is 78%, the highest in Europe, and one parent would, therefore, have to stay at home from work to take care of the child in most families [15].

The number of days with analgesics after surgery was multiplied by (5/7 = 0.71429) to obtain the number of missed working days for the caregiver, as most people work 5 of 7 weekdays. Cases with prolonged use of analgesics due to complications such as infections and re-operation are included in the data, as the follow-up time is 30 days after surgery and most of these cases will be resolved during that time period. According to data from the Swedish Social Insurance Agency, the male-to-female ratio of parental absenteeism is 37 vs 63% and the same distribution was used in our calculations [15]. The indirect costs related to each surgical method (TE, TE + A, TT, TT + A) were calculated separately, depending on the individual mean days with analgesics postoperatively.

### Social insurance agency data

In Sweden, a caregiver is entitled to temporary parental benefit from the government through the Social Insurance Agency, as compensation for salaries lost when staying home from work to care for a sick child. Days absent from work are reported to the Social Insurance Agency (http://www.forsakringskassan.se). In Sweden, all individuals have a unique social security number, used both in the NTSR and in the Social Insurance Agency database. By matching the children’s social security numbers from the NTSRS with those from the Social Insurance Agency, information on the number of days parents received temporary parental benefits during the month following surgery was collected. As benefits are only paid for the days people are actually supposed to work, these were the exact number of days that the caregivers stayed home from work and no correction was needed to exclude weekends in these calculations.

### The human capital approach

Indirect costs due to caregiver absenteeism were calculated according to the human capital approach (HCA) [16]. According to the human capital approach, the value of time lost from work due to a child’s illness is directly related to the expected earnings during that period, which means that 1 day of lost productivity is equal to 1 day of salary plus social costs. The input data are presented in Table 1. Information on average income for men and women in Sweden in 2014 was collected from Statistics Sweden [17], as these were the most recent statistics available. The additional cost of payroll taxes (41.9%) was added, according to information from the National Institute of Economic Research (NIER) [18]. No consideration was taken of differences in salaries between urban and rural areas, as the differences in average salaries between large cities, small cities and rural areas were generally small.

**Table 1:** Input data relating to indirect costs and production losses

Variable	Value (SEK)	Value (EUR)
Average salary per month for men in 2014 (Statistics Sweden) [15]	33,600	3694
Average salary per month for women in 2014 (Statistics Sweden) [15]	29,200	3210
Production cost per month including payroll taxes, 41.9%, for men in 2014	47,678	5241
Production cost per month including payroll taxes, 41.9%, for women in 2014	41,435	4555
Average number of working days per year in Sweden (NIER) [16]	251	28
Average production cost per day for men in 2014	2279	251
Average production cost per day for women in 2014	1981	218
Average production cost per day in 2014 with the distribution of 37% for men and 63% for women who receive temporary parental benefit to care for children according to the Swedish Social Insurance Agency [13]	2091	230

Salary costs were calculated to estimate the cost of production losses according to the human capital approach (HCA) [14]. All costs were calculated in SEK in 2014 prices and transformed to euros using the average exchange rate in 2014 of EUR 1 = SEK 9.0968

All costs were calculated in SEK in 2014 prices and transformed to euros, using the average exchange rate in 2014 of EUR 1 = SEK 9.0968, according to Sveriges Riksbank, the Bank of Sweden (http://www.riksbank.se).

### Statistical analyses

Descriptive analyses including frequencies, mean, minimum and maximum, as well as a one-sample t test with calculations of 95% confidence intervals (CI), were conducted using IBM SPSS statistics 21.

This study was approved by the local ethical review board at the University of Gothenburg, Dnr 885-12 and 594-10.

## Results

**Fig. 1:**
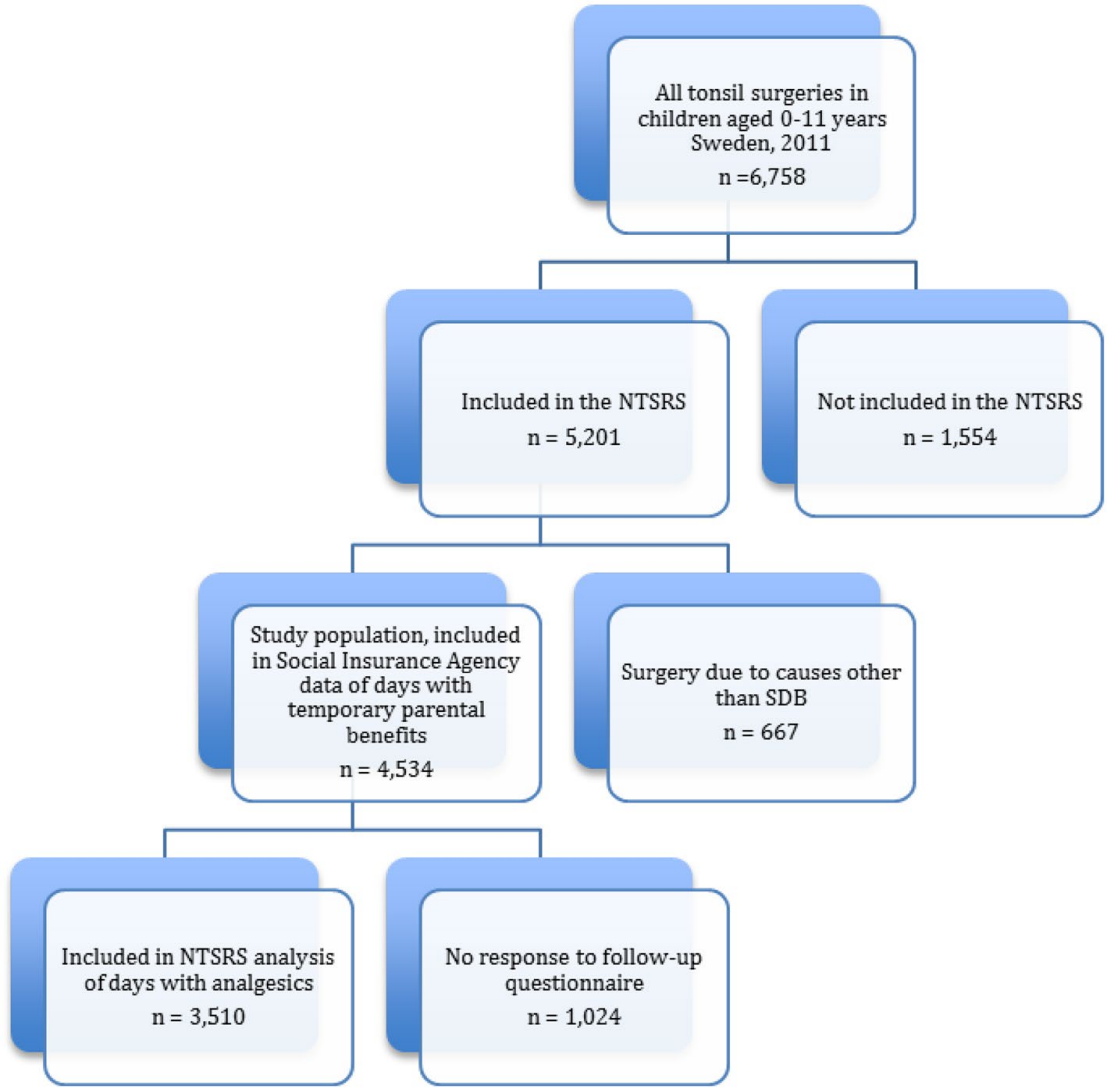
Flow chart showing the selection of the study population

A total of 5201 tonsil surgeries in children aged 1–11 were reported to the NTSRS for 2011. Of these, 4534 (87%) underwent tonsil surgery because of obstructive upper airway symptoms, snoring and/or tonsil hypertrophy and were included in the study. Data regarding temporary parental benefits were collected for all these children. Of the 4534 children, 1024 did not answer the question regarding the use of analgesics in the follow-up questionnaire after 30 days and the main analysis is thus based on 3510 children, Fig. 1.

Table 2 shows the gender and age distribution of the children and the calculated indirect costs due to caregivers’ absence from work after surgery. The mean duration of analgesic treatment was 4.6 days at an indirect cost of EUR 747 and the mean number of days with temporary parental benefits was 2.9 (EUR 667). In Table 3, the indirect costs due to caregivers’ absenteeism are shown for TE, TE + A, TT, and TT + A, respectively. Ten patients were excluded from this analysis, as the type of surgery was inconsistently registered.

**Table 2:** Average indirect cost per patient calculated from register data in 2011 according to the gender and age of children (EUR, 2014 prices)

	Number of patients *N*	Days with analgesics (min–max)^a^	Indirect costs (EUR)	Days with temporary parental benefits (min–max)^b^	Indirect cost (EUR)
Gender of children					
Male	2634	4.5 (0–30)	744	2.9 (0–23)	663
Female	1900	4.6 (0–30)	750	3.0 (0–24)	691
Age of children					
1	93	4.1 (0–14)	667	3.4 (0–24)	771
2	524	4.6 (0–30)	755	3.3 (0–23)	755
3	994	4.4 (0–30)	714	3.1 (0–23)	705
4	964	4.4 (0–25)	724	3.0 (0–21)	685
5	701	4.6 (0–30)	757	2.8 (0–17)	638
6	402	4.9 (0–17)	798	2.8 (0–19)	654
7	286	4.5 (0–14)	740	2.7 (0–14)	629
8	207	4.7 (0–21)	773	2.6 (0–11)	601
9	154	4.6 (0–21)	754	2.6 (0–12)	594
10	107	5.0 (0–14)	824	2.5 (0–13)	578
11	102	5.5 (0–14)	901	2.7 (0–14)	620

^a^Days with analgesics are multiplied by 5/7 to eliminate weekends from the calculation

^b^The reported number of days with temporary benefits, the actual working days that have been compensated for by the Social Insurance Agency. So, no calculations are needed to exclude the weekends

**Table 3:** Average number of days with absence from work for caregivers and production loss related to type of surgery based on register data in 2011 (EUR, 2014 prices)

Type of surgery	*N*	Days with analgesics^a^	95% CI	Indirect costs (EUR)	Days with temporary benefits^b^	95% CI	Indirect costs (EUR)
Tonsillectomy	273	6.2	5.50–6.80	1010	3.5	3.07–4.00	813
Adenotonsillectomy	1117	6.3	5.96–6,61	1031	3.5	3.24–3.71	799
Tonsillotomy	429	3.8	3.46–4.19	629	2.5	2.18–2.73	564
Tonsillotomy + adenoidectomy	2705	3.8	3.65–3.97	626	2.7	2.61–2.84	627
Total	4524	4.6	4.46–4.69	747	2.9	2.84–3.03	674

^a^Days with analgesics are multiplied by 5/7 to eliminate weekends from the calculation

^b^The reported number of days with temporary benefits, the actual working days that have been compensated for by the Social Insurance Agency. So, no calculations are needed to exclude the weekends

The indirect costs related to TE and TE + A were 61% higher than those for TT and TT + A, measured as days with analgesics. This is explained by a recovery time that was 2days shorter after TT compared with TE. The adenoidectomy did not contribute any additional recovery time.

To calculate a total indirect cost due to absenteeism after surgery, it is possible to extrapolate the results of the 4534 operations included in the study, representing 78% of the total number of surgeries due to SDB symptoms in 2011, compared with the 5817 surgeries performed in total. The total indirect cost of absenteeism would then be estimated at EUR 4.34 million per year in Sweden.

## Discussion

The results show that the mean cost of caregiver absenteeism after a child’s tonsil surgery because of sleep-disordered breathing is EUR 747. The choice of surgical method affects the indirect costs, favouring the use of TT over TE due to less postoperative pain.

In this study, two methods were used to estimate absenteeism after tonsil surgery based on the national database, the National Tonsil Surgery Register in Sweden. The reported number of days the child needed analgesics was used as a proxy for the minimum numbers of days the child had to stay home after surgery. This proxy is feasible in the Swedish setting, where most children aged 2–11 in Sweden either attend day-care centres or schools where no analgesics are given by the staff and one caregiver would therefore, have to stay home with the child during this period. It is, however, possible that children stay home longer than analgesics are needed and in fact some surgical centres in Sweden recommend 7–10 days’ absence from day-care centre or school after surgery. In Sweden, all working caregivers who need to stay home for a child’s sickness are entitled to temporary benefits. Data were, therefore, also gathered from the Social Insurance Agency for the same children regarding temporary parental benefits in the 30 days following surgery. The costs of caregiver absenteeism were slightly less when measured with this method. This may also be an underestimation of the true absenteeism, as it does not include certain groups such as students, parents on parental leave or those who are unemployed. Parties other than caregivers could also have been involved in the postoperative care of the child. This could reduce the actual productivity loss. It is interesting, however, that the two methods show a number of days of recovery that is in accordance with previous studies of tonsil surgery for SDB.

The children received analgesics for 6.2 days after a tonsillectomy (TE) and 3.8 days after a tonsillotomy (TT), which is similar to what Walton et al. found in a meta-analysis of 10 studies comparing the return to normal daily activities after paediatric TE and TT (6.6 vs 4.1 days) [19]. The finding that the recovery time is shorter after a TT than after a TE is in agreement with previous studies and is directly related to less pain and a shorter healing process [3, 19, 20]. Another explanatory factor is that TT is known to carry a considerably lower risk of secondary bleeding than TE [3], which would, in turn, reduce absenteeism in the first weeks after surgery.

Recent meta-analyses have shown tonsillotomy to be advantageous in terms of postoperative pain and risk of postoperative bleeding compared with tonsillectomy, with no significant differences in relieving obstructive symptoms, quality of life or postoperative immune function [21–23]. Although the total risk for tonsillar regrowth or need for secondary surgery is only 2–4%, it is considerably higher after TT than TE [23, 24]. Odhagen et al. have shown that the mean risk for secondary surgery is 7 times higher for TT compared to TE, with increasing risk with lower age at first surgery [24].

From a long-term perspective, TT is, therefore. likely to be associated with more indirect costs due to a higher rate of re-operation than TE, but these questions go beyond the scope of this study. Large randomised controlled trials with long-term data are needed to determine whether tonsillotomy is capable to replace tonsillectomy to resolve upper airway obstruction resulting from tonsillar hypertrophy.

In this study, the direct costs of surgery have not been analysed. This has been done in other studies showing a large variation in direct costs between different health care facilities, depending on numerous factors [12, 25]. In one study, the cost of using radiofrequency was 8 times higher than for monopolar cautery in pediatric adenotonsillectomy [26]. Also, coblation tonsillectomy has been shown to be considerably more expensive than electrocautery due to high costs for disposables, despite a shorter time in the operating theatre [27] .This important aspect needs further analysis, especially since tonsillotomy is usually performed with more expensive surgical methods than tonsillectomy [28]. The KPP database is a national Swedish database (http://www.skl.se) that includes the costs of different surgeries from 47 different hospitals in Sweden. The database reveals that tonsil surgery in day surgery costs EUR 1000–1500 and EUR 2800–3000 in inpatient care [29]. More than 74% of paediatric tonsil surgeries in Sweden are now performed in day surgery [30]. The mean cost of the absenteeism after tonsil surgery for SDB in day surgery would thus be 33–43% of the total cost.

The data used in this study were based on the National Tonsil Surgery Register in Sweden (NTSRS), which has, in turn, been validated against the National Board of Health and Welfare’s register of all surgical procedures performed in Sweden with diagnosis and surgical code, date of surgery, and the social security number of the patients [30]. These large national registers enable easy access to information relating to a large number of patients. Another advantage is that the data in the NTSRS regarding indication and surgical procedure were entered by the operating surgeon, which should reduce the risk of misclassification. Further, the follow-up questionnaire that was answered by the caregiver after surgery was administered by the NTSRS, which is unrelated to the clinic where the child had the surgery. This should reduce the risk of bias related to the doctor–patient relationship.

This study has some weaknesses that are needed to be addressed. In addition to the risk of underestimating absenteeism due to the reasons explained above, not all tonsil surgeries in Sweden are included in the NTSRS and 22% of all procedures are not registered. Moreover, 23% of the subjects included did not respond to the follow-up questionnaire. This creates the possibility of selection bias. The possibility of recall bias when answering the questionnaire 1month after surgery should also be taken into account. Although national guidelines exist [14], there may be local variations in the choice of surgical methods. It is also possible that the tonsillectomy group differs from the tonsillotomy group in the aspect that they have probably had more infections prior to surgery and that may affect the post-operative pain and recovery time. Though days with analgesics indicate the level of postoperative pain which would be comparable between children in different countries, differences in national reimbursement systems and family organisation in relation to working parents could affect the final costs imposed on society in different countries. In spite of this, our results agree well with those in studies from other countries regarding the postoperative recovery of the children in the study.

When estimating the total cost of tonsil surgery due to SDB in children, it is important to take account of the increased costs that SDB has due to the increased use of health-care services, as well as the indirect costs due to caregivers staying home to take care of their sick children [8, 10]. TT or TE for upper airway obstruction in children with sleep-disordered breathing (SDB) has been shown to improve general well-being, quality of sleep and cognitive function in several studies. If left untreated, SDB may cause significant morbidity and a reduction in quality of life [31]. In the light of this, the indirect costs associated with surgery found in this study appear reasonable. Our results also show that the choice of surgical method affects the costs, advocating the use of tonsillotomy over tonsillectomy.

## Conclusions

The results show that the mean cost of caregiver absenteeism after a child’s tonsil surgery because of sleep-disordered breathing is EUR 747. The choice of surgical method affects the indirect costs, favouring the use of tonsillotomy over tonsillectomy for the treatment of children with SDB, due to less postoperative pain.
